# The first evidence of a new genotype of nephropathogenic infectious bronchitis virus circulating in vaccinated and unvaccinated broiler flocks in Algeria

**DOI:** 10.14202/vetworld.2018.1630-1636

**Published:** 2018-11-29

**Authors:** A. Lounas, K. Oumouna-Benachour, H. Medkour, M. Oumouna

**Affiliations:** 1 Department of Medicine and Animal Surgery, Veterinary Sciences Institute, University of Blida, Algeria; 2 Department of natural sciences and life, Faculty of Sciences and Technology, University Yahia Fares of Medea, Algeria; 3 Microbes, Evolution, Phylogeny and Infection, UMR Aix-Marseille University, IRD, APHM, IHU Méditerranée Infection, Marseille, France

**Keywords:** avian infectious bronchitis virus, broilers, kidney damages, Algeria, hemagglutination inhibition test, reverse transcriptase-polymerase chain reaction, IBDZ13a

## Abstract

**Background and Aim:**

Avian infectious bronchitis virus (IBV) frequently infects broilers and is responsible for severe economic losses to the poultry industry worldwide. It has also been associated with kidney damage in the broiler flocks. The aim of the present study is to determine the presence of IBV and its possible involvement in kidney damage of broiler chicks.

**Materials and Methods:**

14 clinically diseased broiler flocks from Western and Central Algeria were sampled and analyzed by hemagglutination inhibition (HI) test and reverse transcriptase-polymerase chain reaction (RT-PCR) followed by phylogenic analysis.

**Results:**

The QX (100%) and 4/91 (60%) IBV serotypes were the most prevalent in the kidney damaged broilers regardless of vaccination status. The molecular detection of avian IBV by RT-PCR identified six samples as positive, of which only two isolates were typable by sequencing. We identified a novel IBDZ13a genotype which showed 93% sequence homology to the partial-S1 gene sequence of the IB 4/91 commercial vaccine strain. Sequencing analysis characterized this virus as a novel and divergent IB 4/91 field virus with eight amino acid substitutions that might have resulted in altered immunogenicity.

**Conclusion:**

The isolation of a new IBV strain (IBDZ13a) from vaccinated broiler flocks may explain the failure of the vaccination programs against IBV field strains. Combination of the HI test and RT-PCR indicated that the nephropathogenic IB outbreaks in broilers are related to this novel strain.

## Introduction

Poultry production in Algeria faces many zootechnical and health constraints, such as viral infections like avian infectious bronchitis (IB). The avian IB virus (IBV), a member of the Coronaviridae family (order *Nidovirales* and genus *Coronavirus*), frequently infects broilers and egg-laying hens and leads to severe economic losses to the poultry industry [1]. Since its discovery in the 1930s [2], the IBV has been identified as the major cause of respiratory infections and poor zootechnical performances. Interestingly, it can also multiply in the renal tissue and cause nephritis [3], a phenomenon first described in the United States [4]. More recently, IBV-associated nephritis has been accepted as the most pressing problem in broiler flocks in many countries [5-9].

The most effective method of protecting poultry from IBV infections is through live or killed vaccines [1]. However, nephritis associated with infectious bronchitis has been observed in several vaccinated flocks, suggesting that the current vaccination strategies against IBV may not provide adequate protection [3,10]. In fact, outbreaks of IB are frequently caused due to the strains serologically different from those used for vaccination [11,12]. Since its discovery in 1931, a large number of serotypes or variants of IBV have emerged [5,13,14], and little or no cross-protection occurs between these serotypes [15]. Therefore, it is crucial to track epidemic-causing serotypes in each geographic region or country and produce new vaccines to control IB.

In Algeria, poultry flocks have been vaccinated against IB with the Massachusetts (Mass) strain combined with the IB 4/91 United Kingdom variant strain or 793/B since the past few years. However, kidney damage with suspicion of IB has also been reported in recent years in spite of vaccination but has not been confirmed so far. This has led to the speculation of the possible emergence of variant strains against which conventional vaccines are not completely effective [16].

The aim of this study was to investigate the presence of IBV among Algerian broiler flocks and its possible involvement in broiler kidney damage. Clinically diseased broiler flocks were sampled and analyzed by the hemagglutination inhibition (HI) test and reverse transcriptase-polymerase chain reaction (RT-PCR) followed by phylogenic analysis.

## Materials and Methods

### Ethical approval

All experimental procedures were approved by the Institutional Animal Care Committee of the National Administration of the Algerian Higher Education and Scientific Research (Ethical approval number: 98–11, Law of August 22, 1998) and were conducted according to the recommendations of the “Guide for the Care and Use of Laboratory Animals.”

### Sample collection

Fourteen commercial broiler flocks from Western and Central Algeria in which the chickens were suffering from kidney damage and respiratory disease were sampled between January and December 2012. Trachea and kidney specimens were taken from 10 diseased chickens from each flock and were directly applied to the FTA^®^ cards (GE Healthcare, UK) during the acute phase of the clinical outbreak. The samples were transported and analyzed at the Laboratory of Virology, Instituto Zooprofilattico Sperimentale Delle Venezie, Italy. In addition, blood samples were also collected from each bird to determine the prevalence of different IBV serotypes. The serum was collected from the samples by centrifugation (2000 rpm/min 5 min) and stored at –20°C for further analysis. HI test was performed at MSD International laboratory, Amman, Jordan.

### RNA extraction from FTA^®^ cards and reverse transcription

Three pieces of 2 mm discs from the spotted area of the filters were cut by a sterile scissor and placed in 1.5 mL Eppendorf tubes. Separate sterile scissors and pincers were used for each sample to prevent cross-contamination. Each disc was placed in 750 µL Trifast^®^ FL (PEQLAB, Germany), homogenized, and incubated for 5 min at RT. The Trifast FL kit (Peq GOLD TriFast™ FL, Peqlab) was used to extract the RNA from the FTA^®^ paper according to the manufacturer’s instructions (PEQLAB Biotechnologie GmbH, Erlangen, Germany). RNA purity and concentration were determined by NanoDrop^®^ ND–1000 (Peqlab Biotechnologie GmbH, Erlangen, Germany). Reverse transcription was performed using the ImProm-II™ Reverse Transcription System (Promega, Madison, WI) with random primers according to the manufacturer’s instructions. Briefly, 0.1 µM lyophilized oligonucleotides provided with the kit were dissolved in 1 mL RNase-free water, and 1 µL of the random primer solution was mixed with 4 µL viral RNA and incubated at 70°C for 5 min. After adding Buffer 5× (56 µL), MgCl_2_ (67.2 µL), dNTP (14 µL), RNAsin (7 µL), ImProm II (14 µL), and H_2_O (51.8 µL), the mixture was incubated at 25°C for 5 min followed by 55°C for 60 min. The reaction was terminated by heating at 70°C for 15 min and cooling on ice.

### PCR amplification of the S1 gene

The primer sets (Oligo5 [forward]: 5’-TGA AAA CTG AAC AAA AGA CA-3’ and CK2 [reverse]: 5’-CTC GAA TTC CNG TRT TRT AYT GRC A-3’) were designed for the IBV S1 gene, which contains the hypervariable region 1 and most of the hypervariable region 2 [17]. The S1 gene was amplified with the Superscript^®^ III One-Step RT-PCR Platinum^®^ *Taq* HiFi (Invitrogen™ Life Technologies™, Carlsbad, CA), which generated a PCR product of 344 bp. For the PCR reaction, the following reaction mix was made: 35 µL Buffer 10×, 14 µL dNTP, 14 µL CK2, 14 µL Oligo5, 243.25 µL H_2_O, 1.75 µL Taq polymerase (*Taq* HIFI, Invitrogen™ Life Technologies™, Carlsbad, CA), and 2 µL cDNA to a final volume of 25 µL. The PCR parameters were as follows: 45 cycles of denaturation (94°C, 20 s), annealing (51°C, 1 min), and polymerization (72°C, 1 min). The pre-denaturation step was done at 94°C for 5 min and post-polymerization step at 70°C for 10 min. A 20 µL aliquot of each PCR product was electrophoresed at 120 V for 40 min in 1.5% agarose gel, which was then stained with GelRed™ (Genaxxon Bioscience, Ulm, Germany, GmbH).

### S1 gene sequencing

The amplified cDNA was purified using the NucleoSpin^®^ (MACHEREY-NAGEL GmbH, Germany) Gel and PCR Clean-up kit according to the manufacturer’s instructions and sent for sequencing to x-OVO Limited (x-OVO Limited Laboratories, Thomson Cooper, United Kingdom). The nucleotide and amino acid sequences of the S1 gene of the IBV isolates were assembled, aligned, and compared with reference IBV strains using BASIC BLAST program in DNAStar. Phylogenetic analysis of the S1 amino acid sequences was performed with the neighbor-joining method using the DNAStar software [18]. The partial S1 nucleotide sequences from the IBV isolates have been submitted to the GenBank database and assigned the accession number MG252734. The nucleotide and amino acid sequences of the S1 gene of IBV strains used for the comparison are available in GenBank under the following accession numbers: CR 88 (KM067900), 4/91 (JN600614), Qx (AF193423), Ma5 (AY561713), H120 (M21970), M41 (GQ219712), D274 (KR106999), Italy 02 (AJ457137), and QX D388 (DQ674739).

### HI test

The specific IBV strains that were used in this study were M–41 (a US strain), 4/91 (European vaccine strain), D274 (a Dutch field isolate), and QX (Chinese strain). HI test was performed using the FLOKSCREEN IB-HI TEST kit according to the manufacturer’s instructions. Briefly, the sera were serially diluted and mixed with an equivalent volume of the antigen. After 30 min of incubation at 20°C, the red blood cells of SPF chickens (anticoagulant solution) were added and incubated further for 40 min at 20°C. The plates were then tilted to observe inhibition of hemagglutination.

### Statistical analysis

Statistical Package for the Social Sciences version 20 (IBM, USA) was used for statistical analysis. The geometric mean titer (GMT) was calculated by counting the number of wells from all serum samples within one flock showing HI activity, and the average number was then cross-checked against GMT values given in Brug’s table. The results were reported as the GMT for a given flock against a particular IBV antigen. Descriptive statistics including percentages, means, and frequency distribution were calculated for each of the variables.

## Results

Information regarding the sampling date, biosecurity status, region of sampling, vaccination programs, necropsic and clinical signs, the age of the broilers, and mortality rates are summarized in Table-1. We collected 14 samples from broiler farms located in Western and Central Algeria from the flocks showing kidney damage and high mortality rates. Eight of these farms had not vaccinated their flocks against IBV, 2 farms conducted the prototype vaccination program but with a short interval between the first and second vaccinations, and 4 farms vaccinated their broilers only against the Mass serotype. All vaccinations were performed through the drinking water route, and all the sampled broiler farms showed poor zootechnical performance with rare period elongation (until 71 days in farm 2) and high mortality rates (up to 50% in farms 11 and 13).

**Table-1:** Farm characteristics and the history of IBV outbreaks in Algeria.

Flock number	Age of sampling (d)	Season of sampling	Time between outbreak-sampling	Region	Biosecurity status	Mortality rate (%)	Clinical signs	Vaccination program
1	51	Spring	20	West	Average	48	Renal, respiratory	No vaccination
2	71	Summer	23	West	Average	13	Renal, respiratory	d7: Ma 5
3	60	Summer	27	Central	Average	26.5	Renal, respiratory	d7: Ma5 d14: IB 4/91
4	56	Spring	16	Central	Average	15	Renal, respiratory	d5: Ma 5
5	52	Summer	14	West	Poor	30.5	Renal, respiratory	No vaccination
6	58	Winter	18	Central	Poor	22	Renal, respiratory	No vaccination
7	65	Winter	25	Central	Average	25	Renal, respiratory, digestive	d5: Ma 5
8	52	Winter	15	West	Poor	24	Renal, respiratory	No vaccination
9	42	Winter	11	Central	Average	25	Renal, respiratory	No vaccination
10	49	Winter	11	Central	Average	14	Renal, respiratory	No vaccination
11	65	Summer	25	Central	Poor	50	Renal, respiratory, digestive	d7: Ma5 d14: IB 4/91
12	38	Spring	7	Central	Poor	26.5	Renal, respiratory, digestive	No vaccination
13	52	Autumn	30	West	Poor	50	Renal, respiratory, digestive	d7: H120
14	55	Autumn	15	West	Average	14.5	Renal, respiratory	No vaccination

IBV=Infectious bronchitis virus

The seroprevalence of various IBV strains in broiler flocks suffering from kidney damage in Algeria is summarized in Table-2. All the broiler flocks (100%) tested in this study were seropositive for the QX variant strain, with high mean titer levels (MT=11 log_2_) regardless of the vaccination status. Furthermore, 60% and 50% of the broiler flocks were seropositive for antibodies against the 4/91 and M–41 strains, respectively, although with low mean titer levels. The seroprevalence of IBV D–274 was the lowest with only 7% of the broiler flocks testing positive.

**Table-2:** IBV-HI antibody titers in broiler flocks suffering from kidney damages.

Flocks	Mean titers (log_2_)				Mean titers range (maximum-minimum) (log_2_)			
	
	Qx	M–41	D–274	4/91	Qx	M–41	D–274	4/91
1	11.00	5.80	4.70	7.00	11—11	4—9	4—9	6—11
2	11.00	7.00	4.64	7.55	11—11	5—10	4—7	7—9
3	9.70	6.50	5.20	7.20	7—11	4—11	4—8	4—11
4	10.82	6.18	5.18	7.00	9—11	5—11	4—9	4—11
5	11.00	8.09	6.91	9.00	11—11	6—10	5—10	7—11
6	11.00	6.70	5.10	6.70	11—11	5—8	4—7	5—9
7	9.78	9.78	8.78	9.89	7—11	8—11	5—11	8—11
8	11.00	7.75	5.25	7.50	11—11	7—8	5—6	7—8
9	10.50	5.33	4.50	5.17	8—11	4—9	4—6	4—9
10	10.14	4.86	4.00	5.00	5—11	4—8	4—4	4—8
11	9.50	8.33	5.83	9.67	7—11	6—10	4—8	7—11
12	8.73	7.55	5.91	8.00	4—11	5—9	4—7	5—11
13	9.80	8.70	6.70	8.80	7—11	6—11	4—11	8—11
14	10.75	9.38	6.75	9.88	10—11	8—11	5—10	8—11

IBV-HI=Infectious bronchitis virus-hemagglutination inhibition

**Figure-1:**
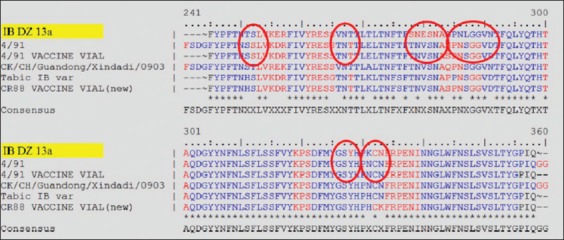
Predicted epitope alignment of deduced amino sequences of HVR S1 gene. New field Algerian isolate and selected infectious bronchitis virus vaccine vial reference strains.

Six of the 14 broiler samples were positive for the IBV S1 gene, but only two isolates were typable by sequencing and showed a high load of IB viral RNA (Ct_1_=22.15 and Ct_2_=31.4). One of the two sequences was identified as the IBDZ13a genotype. Furthermore, IBV was only detected in the kidney tissues and not in the trachea. All samples showed 93% homology to the partial-S1 sequence acquired from the IB 4/91 reference strain (Figure-1). The IBDZ13a strain was characterized as an IB 4/91 variant IBV with 29 nucleic acid substitutions differing from the known IB 4/91 vaccine reference strain sequence, which corresponded to eight amino acid substitutions relative to this reference strain (Table-3). Three of these amino acid substitutions were from a hydrophobic to a hydrophilic amino acid. A change in hydrophilicity altered the epitopes on the S1 protein of the IB DZ13a virus. The predicted epitopes with differences between the field virus and IB 4/91 vaccine strain are shown in Figure-1.

**Table-3:** Changes in IBDZ13a amino acid sequence relative to the known IB 4/91 vaccine strain sequence.

Position	Vaccine strain sequence	Hydrophobicity index	Sample sequence	Hydrophobicity index	hydrophobic/hydrophilic switch
253	Alanine	1.8	Threonine	–0.7	Yes
258	Aspartate	–3.5	Glutamate	–3.5	No
261	Valine	4.2	Isoleucine	4.5	No
273	Glutamate	–3.5	Threonine	–0.7	No
286	Proline	–1.6	Serine	–0.8	No
289	Serine	–0.8	Leucine	3.8	Yes
296	Valine	4.2	Glutamine	–3.5	Yes
305	Serine	–0.8	Aspartate	–3.5	No

IB=Infectious bronchitis

## Discussion

Infectious bronchitis has had a devastating effect on broiler farms since the worldwide emergence and increased prevalence of IBV [11,13]. Due to the high rate of mutation and recombination in coronaviruses, new serotypes and genotypes of IBV frequently emerge and have been reported in different regions around the world [11]. More recently, IBV has been associated with nephritis which is now a major problem in broiler flocks worldwide [19-21]. Similar concerns have been reported in Algeria, but no study has been performed so far on IB-associated nephritis [22].

The present study was carried out to investigate the presence of IBV in broiler flocks and its possible involvement in kidney damage. We also monitored the seroprevalence of different IBV serotypes by the HI test and analyzed the phylogenetic characteristics of the new IBV strains isolated in Algeria in 2013 by sequencing. The HI test and virus neutralization test (VNT) are the most commonly used tests for detecting IBV antibodies [9,23]. Studies show extensive cross-reactions between the IBV serotypes detected by the HI test and similar antigenicity detected by the VNT and HI test [24-26]. However, since VNTs are not cost-effective for routine examination, HI tests are commonly used for routine serology [27].

The most frequently detected IBV serotypes in the broiler flocks with nephritis were IB-QX (100%) and IB 4/91 (60%), which have been detected for the 1^st^ time in Algeria by our group. After reporting the nephropathogenic form of IB [3], the above serotypes have been reported in China, Europe, and Africa and have been seen to be associated with severe kidney damage, high morbidity, and mortality in the vaccinated flocks. At present, these serotypes are the predominant field strains in most poultry farms worldwide [13,28]. The serological analysis showed significantly lower titers of IB 4/91 compared to IB-QX, which could be related to the vaccination of some flocks with the IB 4/91 strain. De Wit [23] reported that many factors influence the level of IBV antibodies, of which the most critical is the presence of immunity at the time of infection. This is consistent with the lower anti-IB 4/91 antibody titers seen in the vaccinated flocks compared to the unvaccinated ones. Another factor that can reduce the sensitivity of HI and thus affect the interpretation of the results is the cross-reaction between the different serotypes, especially when multiple serotypes of IBV are involved. We detected cross-reaction between the QX and 4/91 serotypes in many flocks, and 4 flocks showed mixed infection of three serotypes (QX, 4/91, and M41). Therefore, the serotype specificity of HI is reduced after reinfection or vaccination, especially when the subsequent serotype is heterologous [23].

**Figure-2:**
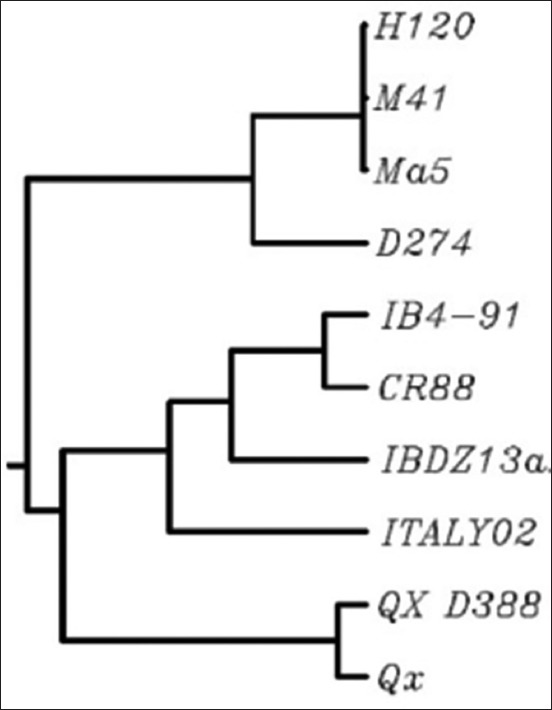
Neighbor-joining phylogenetic tree with 1000 bootstrap replicates expressing the relations among deduced S1 amino acid sequences of IBDZ13a with selected infectious bronchitis virus reference strains.

The S1 protein of IBV determines the genotype, serotype, and phenotype of IBVs [29]. The S1 gene of the IBDZ13a field strain was most closely related to that of the IB 4/91-type strain (Figure-2) as determined by the criterion of Worthington *et al*. [13] and Kannan *et al*. [30], who classified IBV sequences with <99% homology to vaccine strains as variants. Our findings indicate the circulation of virulent strains of IB 4/91, which have become the predominant genotype in Africa, Asia, and Europe [28]. However, we did not see a strong correlation between the HI results which showed the circulation of the QX and 4/91 serotypes and RT-PCR which showed the emergence of a new genotype of the 4/91 strain. This is consistent with the findings of Kusters *et al*. [31] who also reported that RT-PCR and HI results were not exactly correlated, wherein different serotypes showed considerable differences (20-50%) in the amino acid sequences of the S1 subunit. HI is used for routine testing of sera from young growing chickens to determine the serotype-specific antibody status of a flock [32]. However, the presence of multiple cross-reactive antibodies against antigenically unrelated strains, as is often seen during IB outbreaks, reduces the serodiagnostic value of HI. Therefore, a combination of serotyping and genotyping is recommended to identify field strains. Based on the results of HI and RT-PCR, we conclude that the IBDZ13a variant of IB4/91 is involved in kidney damage of broiler flocks.

When live-attenuated and inactivated vaccines of IBV were first introduced, they were effective in controlling IB among poultry flocks [33]. However, vaccinated flocks in many regions of Algeria have shown considerable mortality and production losses in recent years. The isolation of a new IBV strain from the vaccinated and unvaccinated flocks can be explained by two hypotheses. The first hypothesis, based on the mutations and amino acid alterations in the S1 epitope, is that the IBV strain evolved to escape the host immune response elicited by the current vaccines [34]. However, this hypothesis is weak since amino acid alterations do not always translate into differences in antigenicity or biological function. Furthermore, the sequence data only provide information regarding the primary structure of the protein and not the secondary and tertiary structures which are vital for its biological function and antigenicity [23]. The second hypothesis is the failure to adopt the vaccination programs to the IBV field strains circulating in the farms. In our study, only two flocks received the combined vaccine of IB Ma5 (at day 7) and IB 4/91 (at day 14). The failure to protect broilers against nephritis could also be related to the shorter inter-vaccination interval of 7 days. Terregino *et al*. [35] demonstrated improved protection against several IB serotypes by vaccinating with two antigenically different IB vaccines given at the interval of 14 days. This approach obviates the need to develop a new IB vaccine for each new emerging serotype. One flock was vaccinated with the Mass vaccine type H120 strain, which, however, did not provide sufficient protection against IBV infection [33,36].

## Conclusion

We identified IBDZ13a, a new nephropathogenic variant of IB 4/91, for the 1^st^ time in Algerian broiler flocks. Its isolation from the vaccinated flocks indicates poor protection afforded by the vaccination programs to the broilers against infection with new IB variants. Further studies are needed to explain the failure of the current vaccines against the new IB4/91 strain and to investigate the cross-protection and pathogenicity of the new genotype.

## Authors’ Contributions

AL and MO planned and designed the whole study. AL and HM collected samples, performed technique, and analyzed the data. KO and MO supervised the project and helped during manuscript writing, crosschecking, and revision. All authors read and approved the final manuscript.
